# Assessing an assessment: The review and redesign of a competency-based mid-degree evaluation

**DOI:** 10.1017/cts.2018.321

**Published:** 2018-11-05

**Authors:** Colleen A. Mayowski, Marie K. Norman, Wishwa N. Kapoor

**Affiliations:** Institute for Clinical Research Education, University of Pittsburgh School of Medicine, Pittsburgh, PA, USA

**Keywords:** Competency based, portfolio, assessment, formative assessment, reflection

## Abstract

**Introduction:**

Little has been published about competency-based education in academic medicine, in particular how competencies are or should be assessed. This paper re-examines a competency-based assessment for M.S. students in clinical research, and “assesses the assessment” 4 years into its implementation.

**Methods:**

Data were gathered from student surveys and interviews with program advisors, and common themes were identified. We then made refinements to the assessment, and student surveys were administered to evaluate the impact of the changes.

**Results:**

Research results suggested the need to improve communication, time the assessment to align with skills development and opportunities for planning, streamline, and clarify expectations with examples and templates. After implementing these changes, data suggest that student satisfaction has improved without any reduction in academic rigor.

**Conclusion:**

The effective implementation of competency-based training in clinical and translational research requires the development of a scholarly literature on effective methods of assessment. This paper contributes to that nascent body of research.

## Introduction

There is a substantial and growing literature on competency-based medical education [1, 2], examining topics such as its theoretical grounding [3], its application to specialties such as internal medicine and gastronenterology [4, 5], and the competencies required of research coordinators and other clinical research team members [6]. However, with a few notable exceptions [7–9] this literature has focused exclusively on clinical education and not on clinical *research* education, or preparation for academic medicine. With the identification of 14 clinical research competencies by the Education and Career Development Key Function Committee of the Clinical and Translational Science Award community [10], competency-based approaches to clinical research training are gaining traction, with a concomitant need to rigorously evaluate their design and efficacy. One particular need is a deeper understanding of how competencies, once identified, are assessed: What measures are used? How are competency-based assessments integrated into the curriculum? How well do they work, and how can they be improved?

This paper describes the Comprehensive Competency Review (CCR), a midpoint competency assessment for students in the University of Pittsburgh’s Master of Science degree in Clinical Research Program first described by Robinson *et al*. [8], and the approach we took to refining the CCR to enhance both efficacy and efficiency, 4 years after its initial implementation.

## Background

The Master of Science in Clinical Research degree program at the Institute for Clinical Research Education (ICRE) at the University of Pittsburgh trains students in the skills, knowledge, and professional norms appropriate for clinical researchers. It offers a specialization in 5 tracks: translational research, comparative effectiveness research, clinical trials research, health services research, and innovation, each led by a track director. All master’s students take an intensive, 9-credit set of core courses as well as track-specific required courses, electives, and thesis credits. The innovation track began in 2017 and had not enrolled any students at the time of this assessment.

Students enter our program at different career stages—for example, they might be medical students, predoc or postdoc trainees, fellows, or junior faculty. Because of the range of student backgrounds and experiences, it was imperative that we provide students with an assessment checkpoint to monitor their progress toward key competencies. The CCR was instituted in 2013 to meet this need. Its goals, then and now, were to (1) provide students with a structured opportunity to reflect on their learning, (2) monitor their progress, (3) provide individualized feedback, and (4) help them plan their academic experiences to accomplish specific mastery goals. As the development and implementation of this formative competency assessment has been thoroughly discussed elsewhere [8], we will describe it only briefly here before discussing how we assessed and refined it.

The CCR is a midpoint assessment that combines the commonly accepted utility of portfolios [11–13] with the principles of metacognition, or thinking about one’s learning. In its initial instantiation, students compiled a portfolio consisting of 3 reflective 250-word essays, an artifact demonstrating their progress toward achieving 11 ICRE-defined competencies [14], and a short reflection describing why this artifact was chosen. For example, a student might present an abstract written for a manuscript to illustrate progress toward competency in written communication, or a data analysis plan to represent progress toward competency in applied analytic techniques. Students assembled these essays and artifacts into a Word document, then scheduled a meeting with their track director and author CAM to discuss their progress, using their portfolio as evidence.

The CCR was administered in this format from March 2013 to January 2017. In late 2016, we elected to review the CCR—to “assess the assessment”—to ensure that it was accomplishing the intended goals and identify areas for improvement.

## Methods

**Fig. 1:**
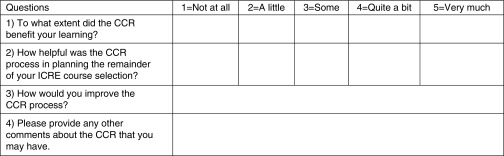
Post-Comprehensive Competency Review (CCR) survey.

We administered an online, 4-question survey to 43 students who completed the CCR before it was revised (Fig. 1). We received 32 responses. Responses were de-identified by the program administrator.

We continued to administer the same survey after making changes to the CCR. In total, 17 students have completed the revised CCR, and 15 responded to the survey.

We also conducted individual hour-long semi-structured interviews with the 4 track directors, whose input was not sought in the development of the initial CCR. Track directors serve as academic advisors to the master’s degree students and participate in every CCR. The authors, one of whom is a trained qualitative researcher, conducted these interviews together. We recorded the interviews, which were transcribed by ICRE staff.

**Fig. 2:**
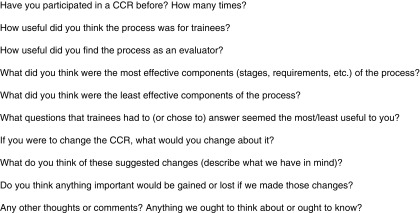
Track director semi-structured interview questions. CCR, Comprehensive Competency Review.

The interview protocol is shown in Fig. 2.

## Analysis

Using a mixed methods approach [15], the authors tabulated the Likert-scale responses from student surveys, then conducted conventional content analysis [16] by hand on the open-ended responses, coding them independently, adjudicating disagreements together, quantifying frequencies, and developing themes.

The authors independently reviewed and coded transcriptions of track director interviews, then met to compare codes, adjudicate differences, and analyze themes. Given that thematic saturation was impossible to reach in such a small sample, we used respondent validation [17], meeting with the track directors as a group to review the themes we identified and verify their accuracy.

## Results

### Student Survey

The majority of students who completed the CCR reported that it benefitted their learning and helped them plan their courses. In response to the qualitative questions, students expressed appreciation for the opportunity it gave them to reflect on the skills they had gained, a process several students described as empowering. They also reported that the feedback they received from track directors during the CCR was valuable to them.

*I think the reflection portion was a nice pause to think about what you feel confident in and what areas you want to gain additional skills…I think it’s helpful for everyone to consciously take the time to evaluate that and ensure the program is really tailored to providing the resources for each person to achieve their individual goals.*

*The feedback was valuable and I would not have performed a thorough self-assessment without the CCR…*

*I thought it was a good reflection exercise and a good way to identify what areas I need to continue to acquire competencies in. I also got some ideas regarding my future studies. Overall it was a positive experience.*

These generally positive findings were consistent with the results reported by Robinson *et al*. [8], and reassured us that the CCR was continuing to serve its pedagogical purpose: to provide an opportunity for students to reflect deeply on their learning, to ensure they were progressing toward acquiring competencies, to receive feedback, and to develop an action plan. However, students also shared critiques and suggestions for improving the CCR in the following areas.

#### Workload

Students reported that the assessment was too writing-intensive, especially on top of their busy academic and clinical schedules. Several complained that it seemed like busywork, particularly the collection of evidence to “prove” competencies. They suggested reducing either the number of competencies addressed or the amount of documentation required.

#### Curriculum

Students expressed concerns that the CCR stood too far outside the rest of the curriculum and was insufficiently integrated with their other academic experiences. One student wrote: *I felt like the CCR process…was a bit isolated from the rest of the ICRE program…As such, it felt like…an arbitrary, task-oriented thing to complete as opposed to flowing logically from the entire program.*

#### Timing

Students expressed concerns with timing. While the CCR was designed to be administered at the midpoint of the degree program (defined as 15 credits), this did not always happen according to plan. Indeed, the lack of a formalized communication process sometimes caused students to schedule their CCR quite late in their degree program, which left them little opportunity to incorporate the suggestions of track directors.

*I was notified to do this quite late in the course of my degree. Thus, my ability to plan future course work based on the CCR process was limited. I think there should be some way to standardize—in terms of credits not time—when the process takes place.*

#### Evidence

Students questioned the request for evidence of every competency. Even when the CCR was administered at the midpoint, as intended, students had difficulty providing evidence of every competency. Although students had strong exposure to 6 competencies emphasized during the required, intensive 9-credit Summer Core, they had limited exposure to the other 5 competencies by the midpoint of their degree program. Often, their evidence for one or more of those 5 was weak and their reflective paragraph discussing the relevance of the artifact was strained and forced rather than a description of authentic learning. As one student expressed it:

*I think for me, part of the difficulty with the CCR process was the timing. Since I was doing just one specific research year, the CCR started very early in the year, so it seemed like I was filling it out without having done the majority of my MS work. I think it could be beneficial if appropriately timed in the middle (or even in the later half) of the training process.*

#### Communication

Students also reported that departmental expectations for the CCR were not communicated clearly. While the CCR’s purpose and process were described briefly during the students’ Summer Core orientation meeting, no other information was formally communicated until the CCR was due. Students often responded with confusion—their orientation was a hazy memory, and they expressed frustration with (as they perceived it) this sudden demand for a time-consuming portfolio project. This suggested that departmental communication about the CCR could be clearer, more consistent, and begin earlier.

#### Examples/Templates

Students frequently requested templates and example CCRs to make it easier for them to discern departmental expectations concerning the style, depth, and length of answers. For example:

*Make the instructions more clear for the competency section of the CCR, e.g. provide anonymous examples. I felt that the instructions were unclear at first and needed to see examples to understand that it was required.*

*I would suggest that students should be given more specific details regarding how to complete the CCR form. Initially I wasn’t sure what to put in the evidence of each competency. A template or a model CCR form could be helpful.*

In total, 52% of the qualitative feedback from students completing the original version of the CCR included suggestions to improve the format and/or implementation of the CCR, indicating to us that refinements were in order.

### Track Director Interviews

Interviews with track directors revealed several themes, most of which dovetailed closely with student surveys. Like students, the track directors thought the CCR provided a valuable opportunity for students to synthesize what they had learned (1 track director described it as akin to a comprehensive exam in that respect). They also believed the CCR helped students approach their learning more reflectively, refine their research projects, and identify training opportunities, as well as to receive individualized feedback on their research ideas and career trajectories.

They had similar concerns as well—for example, the need for appropriate timing. Like many of the students, several directors thought the CCR was taking place too late for the feedback provided to be optimally useful for students. The directors agreed that the CCR should take place around the 15-credit mark to provide the right balance between training completed and training still to come.

Track directors also expressed their concern that students were required to provide evidence of competencies they could not realistically expect to have acquired at the midpoint. This sometimes led students to be perfunctory rather than thoughtful in their analysis. In a similar vein, track directors were asked to grade each artifact “Competent” or “Needs Additional Work,” which was problematic, since “Needs Additional Work” implied that students had exposure to all 11 competencies, which might not be true at degree midpoint. (The grade of “Competent” also raised concerns with at least one track director, who pointedly stated that if a student was competent at the midpoint, why would the student need to complete the remainder of the degree program?) This concern corresponded closely to what we had heard from students and highlighted an interesting tension: if the assessment comes too late, students do not have sufficient opportunity to act on the feedback they are given, but if it comes too early, they do not have sufficient opportunity to produce evidence of competencies gained. This insight helped us to adjust the structure and timing of the assessment, so that the CCR more appropriately targeted both formative and summative goals. It inspired the most drastic change to the CCR: rather than expecting students to provide evidence of progress in all 11 ICRE competencies, we required evidence of exposure to only the 6 we knew were emphasized in the required 9-credit Summer Core. This also inspired our decision to change the grading structure to “Does Not Meet Expectations,” “Meets Expectations,” and “Exceeds Expectations.” A student who receives a grade of Meets Expectations is understood to have achieved a level of competency acceptable for someone who is at the midpoint of a degree program. These changes are more fully described in Table 1.

**Table 1:** Rationale and changes based upon student and track director input

Rationale	Change
Alleviate student confusion about the CCR process	Provide an orientation tailored to incoming clinical research master’s students, explaining the CCR and how it works. Communicate earlier and more often with students
Reduce time burden of writing and assembling the CCR	Email students at the beginning of every semester to remind them to collect evidence and prepare for the CCR as they proceed through their courses
Ensure CCR requirements are integrated more effectively into their degree program and appropriate for their developmental stage	Require artifacts demonstrating only the 6 competencies students could reasonably be expected to have attained at the midpoint of their degree program (not all 11 competencies)
Time CCR to ensure that students have opportunities to integrate formative feedback from advisors	Schedule the CCR earlier in students’ careers to ensure that it falls at the midpoint
Foster intention-setting and forward planning as well as backward reflection	While reducing the number of competencies for which students should demonstrate mastery at the midpoint (see above), require students to choose 3 of the remaining 11 competencies and write an action plan for how they will acquire each
Maintain rigor while eliminating busywork	Require students to come to their advisor meeting prepared to speak to *all* 11 competencies, either by demonstrating mastery *or* by planning a strategy for developing competence. Do not require them to write about all 11 competencies
Raise performance standards by making expectations clearer and eliminating extraneous cognitive load [18]	Provide templates to guide students’ writing as well as models of excellent student CCRs (de-identified and with permission from the writer). Just as seeing examples of successful grants facilitates grant-writing, models would clarify expectations regarding style, length, and tone, without reducing the need for rigorous, independent thought

CCR, Comprehensive Competency Review.

Another shared theme was the need to better integrate the CCR with the rest of the students’ experiences. To this end, directors expressed a desire to have mentors more involved with the CCR to ensure that the feedback and advice given by both mentors and track directors were consistent and focused on competencies.

The final theme among track directors was the desire to ensure that the CCR fostered deep reflection and was not just an exercise in “checking off boxes.” In particular, they were concerned about students incorporating artifacts from coursework without thinking very hard about the competencies they were meant to illustrate, and students charting an action plan that simply replicated the upcoming curriculum. The track directors wanted to ensure that the CCR was implemented in a way that ensured that students were never simply going through the motions of reflection and planning, but were instead engaged in deep, critical thinking.

### Refinements to the CCR

We used the survey and interview data to refine the CCR. Our goal was to address areas of concern without changing the reflective, metacognitive nature of the assessment or compromising rigor. The changes we made and our rationale for each are summarized in Table 1.

## Discussion

In February 2017, we rolled out the refined version of the CCR. Early data suggest that the refined CCR has been well received. Seventeen students have completed the refined CCR and 15 have completed the survey (88%), and we have seen no critiques about timing, communication, or “busywork.” In addition, Likert-scale responses to questions 1 and 2 confirm the CCR’s continuing usefulness, and just one student provided suggestions to improve the implementation. More CCRs are being scheduled at the midpoint (15 credits), allowing students and track directors greater opportunity to discuss thesis options and ideas.

These preliminary results are encouraging, and we predict we will continue to see greater student satisfaction without a compromise in rigor. We will continue to survey students, and will periodically examine the data to ensure that the CCR is serving its intended pedagogical purpose and that the rigor of the assessment is maintained.

## Conclusion

Assessment is an iterative process and even successful assessments should be periodically reviewed and revised. Our evaluation of the CCR 4 years into its implementation has reinforced our original determination that a mid-degree competency-based assessment helps students develop stronger metacognitive skills and habits, along with a keener focus on mastery, self-regulation, and planning. It can also help departments assess students’ progress and provide feedback and direction when it is most useful. However, when such assessments stand outside of an already rigorous formal curriculum, departments would be wise to streamline the assessment process thoughtfully, making efficient use of students’ limited time and energy, while preserving the intellectual value of the assessment. As competency-based clinical research education is still very much in its infancy, we see great value in sharing approaches and lessons learned, so that institutions embarking on competency-based educational approaches can learn from one another and continue to refine and improve the quality of the training they provide.
